# Ropinirole-Associated Orthostatic Hypotension as Cause of a Prescribing Cascade in an Elderly Man

**DOI:** 10.7759/cureus.15506

**Published:** 2021-06-07

**Authors:** Ana F Becerra, Marisa Boch, Yahya A Al-Mezrakchi

**Affiliations:** 1 Department of Medicine, University of Connecticut, Farmington, USA; 2 Department of Internal Medicine, University of Connecticut School of Medicine, Farmington, USA; 3 Department of Internal Medicine, Saint Francis Hospital, Trinity Health of New England (NE), Hartford, USA

**Keywords:** ropinirole, hypotension, side effect, prescribing cascade, older adult, medication safety

## Abstract

Ropinirole is an overall well-tolerated antiparkinsonian medication that is also used to treat restless leg syndrome (RLS). The incidence of side effects is low, with orthostatic hypotension (OH) only anecdotally reported. Additionally, it is known that the elderly population is very susceptible to adverse drug effects and the prevalence of prescribing cascades that these can trigger is unknown.

A 71-year-old male with history of atrial fibrillation, well-controlled diabetes on oral agents, hyperlipidemia, hypertension, ischemic heart failure status post (s/p) implantable cardioverter-defibrillator (ICD) placement with improved ejection fraction (EF), transient ischemic attack (TIA), rheumatoid arthritis, RLS, aortic stenosis s/p mechanical aortic valve replacement on anticoagulation, deep venous thrombosis (DVT), and right knee replacement, presented to the ED with generalized weakness, with difficulty standing from seated position, followed by a fall without head trauma. Over the eight months prior to this presentation, the patient had had similar symptoms that resulted in four falls, two hospital admissions, and new prescriptions of midodrine and compression stockings.

On admission, vital signs were remarkable for positive orthostatics with blood pressure (BP) 110/74 mmHg, heart rate (HR) of 86 bpm in supine position and BP 87/51 mmHg, HR of 70 bpm while in standing position. Physical exam was unremarkable except for a known ejection murmur and dry oral mucous membranes. Labs included a creatinine 3.6 mg/dl, blood urea nitrogen (BUN) 66 mg/dl, international normalized ration (INR) of 4.1, B-natriuretic peptide (BNP) of 313 pg/mL, troponin <0.03 ng/mL. A kidney ultrasound was normal, and a transthoracic echocardiogram showed left ventricle ejection fraction (LVEF) of 55-65%, improved compared to a prior study. Furosemide, carvedilol and canagliflozin were discontinued and IV fluids were administered. In the subsequent days, his creatinine improved, and so did the patient's volume status, but he continued to be orthostatic despite midodrine and stockings. On further interview, the patient disclosed starting ropinirole 0.25 mg three times daily approximately 10 months prior to this admission, due to asymptomatic RLS that was reported in a sleep study. Decision was made to discontinue this medication, which resulted in improvement of symptoms. We were able to discontinue IV fluids, midodrine and stockings, and reintroduce carvedilol, furosemide and canagliflozin in a stepwise manner. In a follow-up visit one month after discharge, the patient was symptom-free.

This case illustrates two major points. First, this prescribing cascade potentially induced by ropinirole, as well as the increase in health care costs associated to iatrogenic admissions, is major preventable problem faced mostly by the geriatric population. Second, although OH associated with ropinirole has only been reported in patients treated for Parkinson’s disease, this side effect should be considered when prescribing ropinirole for other indications, with cautious assessment of risks and benefits. Further studies need to be conducted to establish the frequency of OH related to ropinirole.

## Introduction

A prescribing cascade is a process by which an adverse drug effect is misinterpreted as a new medical condition, resulting in a new, potentially unnecessary, medication being prescribed to treat this new condition. Since the prescribing cascade was first described in 1995, the concept has been expanded to include unnecessary over-the-counter medications and medical devices [1,2]. More than 20 common prescribing cascades have been documented in literature. Examples range from antipsychotics causing drug-induced parkinsonism and prescription of antiparkinsonian medications to anticholinesterase inhibitors leading to gastrointestinal symptoms and self-medication with over-the-counter bismuth subsalicylate. Prescribing cascades are a public health problem that lead to avoidable adverse health outcomes and unnecessary costs for individuals and the health care system.

Ropinirole is one of the Food and Drug Administration (FDA)-approved drugs indicated for the management of both Parkinson’s disease and restless leg syndrome (RLS). Ropinirole and other D2 agonists act by binding with high affinity to post-synaptic D2 dopamine receptors in the central and peripheral nervous systems, simulating the effects of endogenous dopamine [3].

RLS is a sensorimotor circadian sleep disorder characterized by an irresistible urge to move the legs, worsening of symptoms with rest or inactivity, partial or complete relief of symptoms with movement, and exacerbation of symptoms at night [4]. The understanding of RLS pathophysiology remains incomplete. Clinical findings and pharmacologic studies have demonstrated improvement of RLS symptoms with dopaminergic agonists and worsening of RLS symptoms with dopaminergic antagonists, thereby providing strong evidence for the role of dopaminergic system dysfunction in RLS. Treatment of the idiopathic form of RLS is primarily pharmacologic. Dopamine D2 agonists, including pergolide, pramipexole, and ropinirole, remain first-line treatment options for RLS. In particular, ropinirole has shown improvement in symptoms, quality of sleep, quality of life, and mood for patients with moderate-to-severe RLS. Once started, ropinirole should be prescribed at the minimum dose necessary for acceptable symptom reduction [5-9].

Ropinirole is generally well-tolerated. Common side effects include nausea, headache, dizziness, somnolence, depression, vomiting, hallucinations, and behavioral changes [7]. On the other hand, the incidence of orthostatic hypotension associated to ropinirole is only anecdotally reported [7,10]. We present a case of an elderly patient with recurrent episodes of symptomatic orthostatic hypotension associated to ropinirole. This case study proposes a prescribing cascade begun by prescription of ropinirole for asymptomatic restless leg syndrome.

## Case presentation

A 71-year-old Caucasian male with history of atrial fibrillation, diabetes, hyperlipidemia, hypertension, ischemic heart failure, implantable cardioverter-defibrillator (ICD) placement (one year prior to this admission), transient ischemic attack (TIA) (six years before), rheumatoid arthritis, RLS, aortic stenosis with remote mechanical aortic valve replacement on anticoagulation, chronic kidney disease, and right knee replacement, presented to the ED with generalized weakness, with difficulty standing from a seating position, followed by a fall without head trauma. He was taking a beta-blocker, warfarin, a loop diuretic, statin, insulin, and acetaminophen.

**Figure 1:**
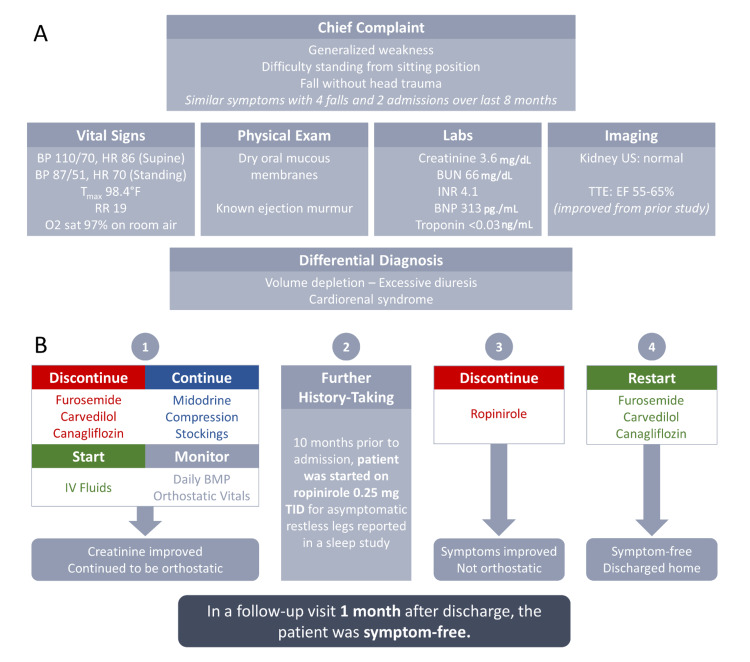
(A) Initial patient presentation and differential diagnosis. (B) Hospital course and follow-up.

Over the past eight months, the patient suffered similar symptoms resulting in four falls and two hospital admissions. During the hospital admissions, he had an extensive workup which revealed orthostatic hypotension. He was treated sequentially with fluids, physical therapy, compression stockings, midodrine and an adjusted antihypertensive regimen (Figure 1).

This time, vital signs were remarkable for orthostatic blood pressure (BP) of 110/74 mmHg, pulse of 86 bpm supine and BP 87/51 mmHg, pulse of 70 bpm standing. Physical exam was unremarkable except for a known ejection murmur and dry oral mucous membranes. Laboratory results included a creatinine 3.6 mg/dL, blood urea nitrogen (BUN) 66 mg/dL, international normalized ration (INR) of 4.1, B-natriuretic peptide (BNP) of 313 pg/mL, troponin <0.03 ng/mL. His INR had fluctuated from supratherapeutic to subtherapeutic for about eight months prior to this admission. Kidney ultrasound was normal (Figure 1). Given the leading differential diagnosis at this time was volume depletion secondary to excessive diuresis, home furosemide, carvedilol and canagliflozin were discontinued, and IV fluids were administered. He was continued on midodrine and compression stockings, which he was using as outpatient. In the subsequent days, his creatinine improved to his baseline, but he continued to be orthostatic. A transthoracic echocardiogram was notable for left ventricle ejection fraction (LVEF) of 55-65%, improved compared to a prior study (Figure 1).

On further interview, the patient reported starting ropinirole 0.25 mg three times daily about 10 months prior to this admission, due to asymptomatic restless legs that was reported in a sleep study performed for obstructive sleep apnea evaluation. Following a shared-decision-making process with the patient, ropinirole and midodrine were discontinued on a step-wise manner. The patient improved his symptoms, and we were able to reintroduce carvedilol, furosemide and canagliflozin. In a follow-up visit one month after discharge, the patient was symptom-free.

## Discussion

**Figure 2:**
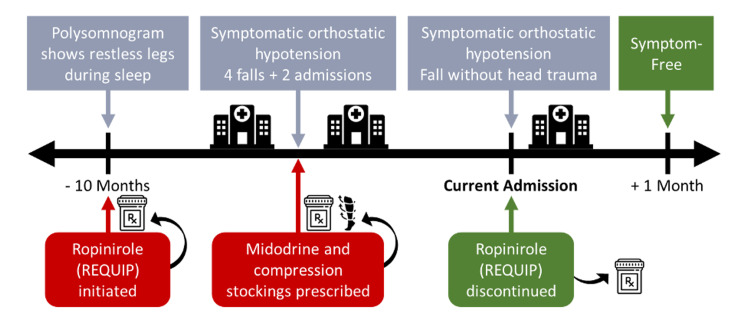
Summary of case

**Figure 3:**
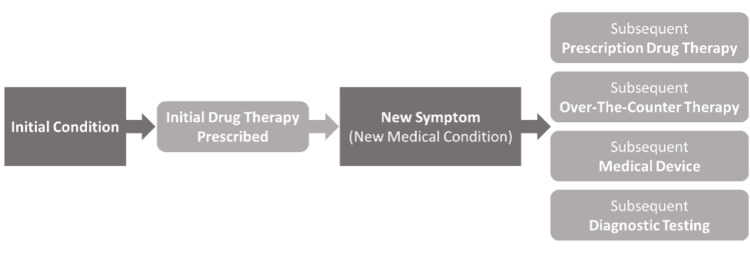
Prescribing cascade overview

In this case, an elderly patient with recurrent episodes of symptomatic orthostatic hypotension and falls being managed with midodrine and compression stockings, was found to have been prescribed ropinirole for asymptomatic RLS approximately two months prior to onset of symptoms. Upon cessation of ropinirole, the patient had resolution of symptomatic orthostatic hypotension up to one month of follow-up post-discharge. Given these clinical findings and literature reports of ropinirol-associated orthostatic hypotension, it is believed that ropinirole use led to the patient's orthostatic hypotension and falls, and initiated the prescribing cascade (Figures 2, 3) [7,10-14].

Dopamine agonists have been shown in clinical studies and clinical experiences to impair regulation of blood pressure, leading to orthostatic hypotension [10]. Patients on dopamine agonists for Parkinson disease are particularly susceptible to postural hypotension, and prevalence has been well-documented in literature with rates of 8.7% to 58.2%. However, the incidence of orthostatic hypotension in patients taking dopamine agonists for RLS is only anecdotally reported. In a phase I study of ropinirole, nine out of 110 (9%) healthy volunteers experienced symptomatic orthostatic hypotension not resulting in syncope or hospitalization, although this was particularly at doses above 0.8 mg. In a phase II study for ropinirole, 14 of 55 patients with RLS (25%) had hypotension or orthostatic hypotension compared to zero of the 27 patients receiving a placebo. In another 12-week placebo-controlled trial of ropinirole, four of 496 patients with RLS (0.8%) reported orthostatic hypotension compared to two of 500 patients (0.4%) receiving placebo [10-13,15-20].

The consequences of the prescribing cascade in this patient include direct costs, such as cost of hospital stays, outpatient clinic visits, prescription medications, medical equipment, and diagnostic workup; and indirect costs, such as time, transportation, diminished quality of life, as well as potential to develop life-threatening conditions due to a fall. In particular, for DRG 312 (Syncope & Collapse) I951 (Orthostatic Hypotension), estimated cost per hospital admission is $32,453.11. For this patient with two hospital admissions secondary to orthostatic hypotension, the admission costs totaled at least $64,906.22.

“Deprescribing” is the process of dose reduction or stopping medication that may be unnecessary or causing excessive harm [1,11,13,14]. In this case, once the medical team realized that ropinirole may be contributing towards the patient’s symptomatic orthostatic hypotension, a discussion with the patient revealed that his RLS was asymptomatic and only identified via polysomnography. In a shared decision with the patient, it was decided that the risks of continuing ropinirole outweighed the benefits, and ropinirole was discontinued.

Of note, the patient's INR fluctuated considerably over the eight months prior to admission. Interestingly, it has been reported that ropinirole may enhance the anticoagulant effect of warfarin. The exact mechanism of this interaction is unknown, but it has been proposed that ropinirole inhibits or competes for CYP1A2, which is partially responsible for R-warfarin metabolism. An alternative mechanism is that ropinirole and warfarin compete for protein binding sites resulting in increased concentrations of unbound warfarin [3,16]. Therefore, given the time line, the supratherapeutic INR that our patient exhibited could have possibly been a result of this interaction.

Because the patient’s orthostatic hypotension resolved following de-prescribing of ropinirole, the benefits of deprescribing evidently outweighed any benefit associated with treating his asymptomatic RLS. In particular, deprescribing would possibly allow this patient to avoid future falls secondary to symptomatic orthostatic hypotension, future hospital stays and outpatient visits, further overprescribing to treat the orthostatic hypotension, and future drug-drug interactions secondary to this overprescribing. It was especially beneficial to decrease this patient’s risk of falls, as he had known fluctuating supratherapeutic INR levels while on warfarin, putting him at increased risk of intracranial hemorrhage and potential death with each fall. By decreasing the patient’s risk of falls and improving his mobility, deprescribing ultimately impacted on his autonomy and quality of life.

Based on these points, the following are key implications for clinical practice. First, in order to avoid adverse effects associated with prescribing cascades and iatrogenic admissions, providers must take deliberate steps to interrupt the prescribing cascade when caring for patients, especially elderly patients. In order to do so, providers should (1) ask if a new drug is being prescribed to address an adverse event from a previously prescribed medication, (2) identify the medication that led to the prescribing cascade, (3) assess if the medication is necessary or if the benefits of continuing outweigh the harm, and (4) reduce the dose or stop the medication. Second, in order to limit orthostatic hypotension induced by ropinirole, providers should carefully weigh the risk and benefits of prescribing ropinirole, caution the use of ropinirole for asymptomatic RLS, and use the lowest dose possible for RLS relief. For providers with patients already taking ropinirole, they should monitor for orthostatic hypotension and falls and reevaluate at regular intervals if the medication dose can be reduced or discontinued (Table 1).

**Table 1:** Proposed recommended actions to avoid ropinirole-associated prescribing cascades RLS: Restless Legs Syndrome.

Before Prescribing Ropinirole	After Prescribing Ropinirole
Weight risks and benefits of prescribing ropinirole for RLS, especially in elderly patients.	Monitor for orthostatic hypotension and falls, especially in elderly patients.
Caution use of ropinirole for asymptomatic RLS.	Determine if: Ropinirole dose can be reduced. Ropinirole can be discontinued.
Prescribe at the lowest dose possible for symptom relief.	

## Conclusions

This case illustrates two major points. First, this prescription cascade caused by ropinirole, as well as the increase in health care costs attributed to iatrogenic admissions, are major preventable issues faced particularly by the geriatric population. Second, although orthostatic hypotension associated with ropinirole has only been anecdotally reported in patients treated for Parkinson’s disease (higher doses), this side effect should be considered when prescribing ropinirole for other indications, with cautious assessment of risks and benefits. Given the little amount of evidence on ropinirole, further studies will need to be done to assess the frequency at which orthostatic hypotension occurs.

Prescribing cascades are an avoidable public health issue that put patients at risk for adverse health outcomes and lead to unnecessary costs for individuals and the health care system. As providers, we must take active steps to interrupt prescribing cascades and therefore must educate ourselves on pharmacologic agents, such as ropinirole, that have the potential to potentiate prescribing cascades. By describing this case of ropinirole potentially contributing to orthostatic hypotension and falls requiring treatment and hospitalization, the hope is that future patients can avoid the consequences of a ropinirole-induced prescribing cascade.
